# Limited grounding-line advance onto the West Antarctic continental shelf in the easternmost Amundsen Sea Embayment during the last glacial period

**DOI:** 10.1371/journal.pone.0181593

**Published:** 2017-07-25

**Authors:** Johann P. Klages, Gerhard Kuhn, Claus-Dieter Hillenbrand, James A. Smith, Alastair G. C. Graham, Frank O. Nitsche, Thomas Frederichs, Patrycja E. Jernas, Karsten Gohl, Lukas Wacker

**Affiliations:** 1 Alfred-Wegener-Institut, Helmholtz-Zentrum für Polar- und Meeresforschung, Marine Geosciences, Bremerhaven, Germany; 2 British Antarctic Survey, High Cross, Cambridge, United Kingdom; 3 College of Life and Environmental Sciences, University of Exeter, Amory Building, Exeter, United Kingdom; 4 Lamont-Doherty Earth Observatory of Columbia University, Palisades, New York, United States of America; 5 Faculty of Geosciences, University of Bremen, Bremen, Germany; 6 University of Tromsø, The Arctic University of Norway, Department of Geosciences, Tromsø, Norway; 7 ETH Zürich, Laboratory of Ion Beam Physics, Zürich, Switzerland; Universita degli Studi di Urbino Carlo Bo, ITALY

## Abstract

Precise knowledge about the extent of the West Antarctic Ice Sheet (WAIS) at the Last Glacial Maximum (LGM; c. 26.5–19 cal. ka BP) is important in order to 1) improve paleo-ice sheet reconstructions, 2) provide a robust empirical framework for calibrating paleo-ice sheet models, and 3) locate potential shelf refugia for Antarctic benthos during the last glacial period. However, reliable reconstructions are still lacking for many WAIS sectors, particularly for key areas on the outer continental shelf, where the LGM-ice sheet is assumed to have terminated. In many areas of the outer continental shelf around Antarctica, direct geological data for the presence or absence of grounded ice during the LGM is lacking because of post-LGM iceberg scouring. This also applies to most of the outer continental shelf in the Amundsen Sea. Here we present detailed marine geophysical and new geological data documenting a sequence of glaciomarine sediments up to ~12 m thick within the deep outer portion of Abbot Trough, a palaeo-ice stream trough on the outer shelf of the Amundsen Sea Embayment. The upper 2–3 meters of this sediment drape contain calcareous foraminifera of Holocene and (pre-)LGM age and, in combination with palaeomagnetic age constraints, indicate that continuous glaciomarine deposition persisted here since well before the LGM, possibly even since the last interglacial period. Our data therefore indicate that the LGM grounding line, whose exact location was previously uncertain, did not reach the shelf edge everywhere in the Amundsen Sea. The LGM grounding line position coincides with the crest of a distinct grounding-zone wedge ~100 km inland from the continental shelf edge. Thus, an area of ≥6000 km^2^ remained free of grounded ice through the last glacial cycle, requiring the LGM grounding line position to be re-located in this sector, and suggesting a new site at which Antarctic shelf benthos may have survived the last glacial period.

## Introduction

**Fig 1:**
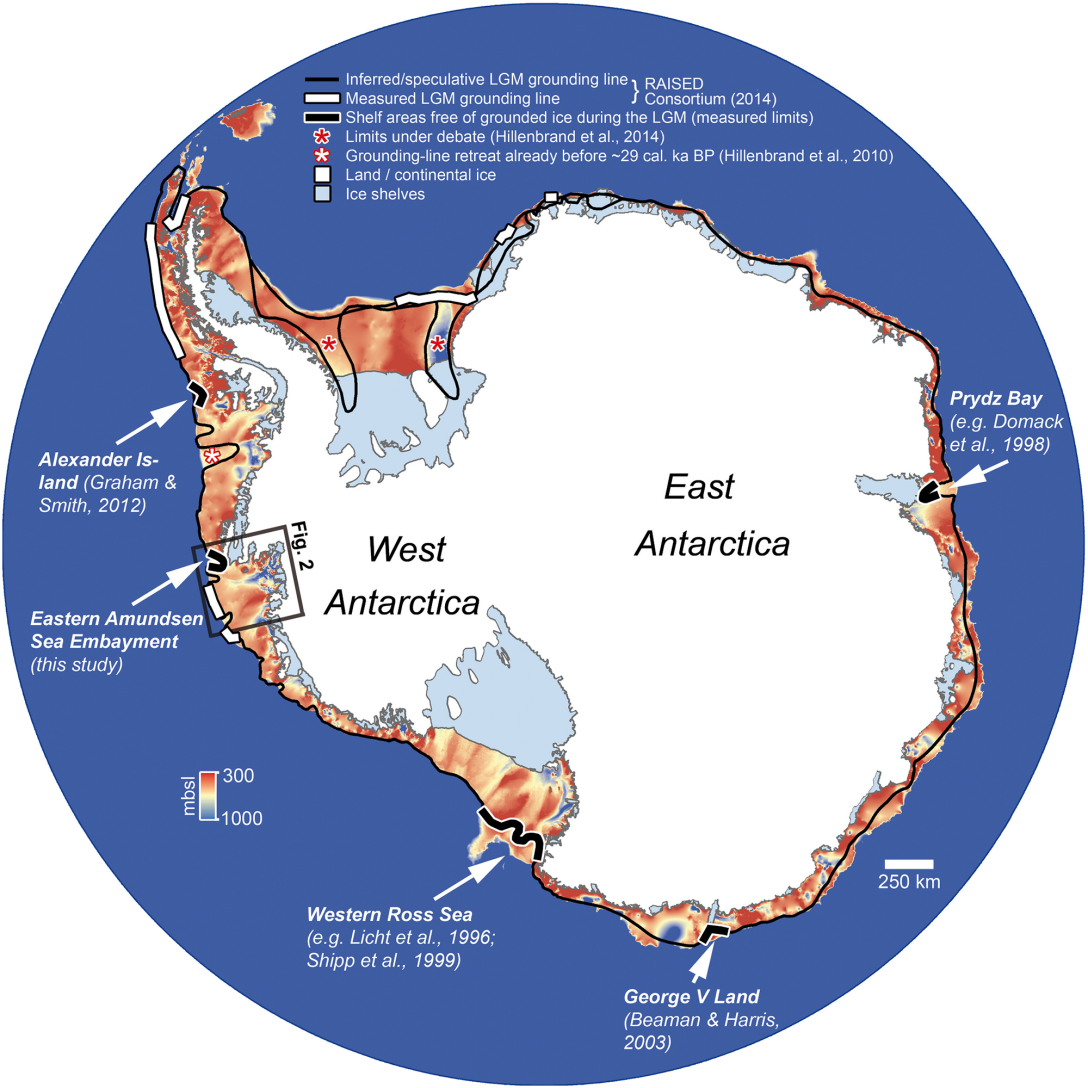
Overview map of presently known locations of grounded-ice free areas during the LGM (thick black lines). Definition of these areas is either based on 1) Landform evidence (George V Land, [19]; Alexander Island [20]), or 2) Landform evidence including geological/dating evidence (western Ross Sea, e.g. [17,26]; Prydz Bay, e.g. [18]; Eastern Amundsen Sea Embayment, this study). The general bathymetry is derived from IBCSO v.1 [27]. The inferred or speculative LGM grounding-line positions are indicated by the thin continuous black line, while measured limits are marked with a thick white line (derived from The RAISED Consortium [4]). The diverging grounding-line positions in the Weddell Sea sector display the currently debated LGM ice limits in this area [28].

During the Last Glacial Maximum (LGM) both geologic data and numerical models suggest that the Antarctic Ice Sheet advanced towards the continental shelf edge in most sectors of the continent (e.g. [1–4]). By integrating glacial geomorphologic, seismic, and sedimentological data collected over past decades from the Antarctic continental shelf, a detailed reconstruction of ice-sheet change during and since the LGM was developed for many shelf areas, with the majority of this work focusing on the Antarctic Peninsula shelf and shelf areas offshore from the West Antarctic Ice Sheet (WAIS) (reviewed by The RAISED Consortium [4]). However, even in those areas records from the shallower outer continental shelf are still sparse, despite this being the key region for defining the LGM grounding line position. The lack of information is primarily due to intense iceberg scouring of the seafloor since or during the LGM and post-LGM current winnowing of surface sediments, which removed pre-existing subglacial bedforms or disturbed the sedimentary record (e.g. [5–7]), but also simply because research vessels often have difficulties to access these outer shelf areas due to intense sea-ice coverage. However, to date, geological and geophysical data used for constraining WAIS extent and subsequent retreat have mainly been acquired from middle and inner shelf regions (e.g. [5,6,8–13]). Therefore, the outer shelf around Antarctica remains one of the least understood environments for ice-sheet reconstructions, and hence still is a major gap in knowledge of Antarctica’s past (e.g. [6,8,10,14]). Consequently, a clear delineation of ice-free areas on the outer shelf during the LGM has not been possible. So far, only a handful of these areas that may have served as potential glacial refugia for benthic communities [15,16] have been identified on the Antarctic shelf (Fig 1) including the western Ross Sea [17], Prydz Bay [18], George V Land [19], and Alexander Island [20]. Identification of these sites is essential in order to correctly constrain the extent of the AIS during the LGM. Furthermore, these sites are needed in order to explain the complex benthic community structure including ancient and endemic species, indicating long-term isolation on the continental shelf over timescales from hundreds of thousands to millions of years (e.g. [21,22]), and thus persistence through multiple glacial cycles.

**Fig 2:**
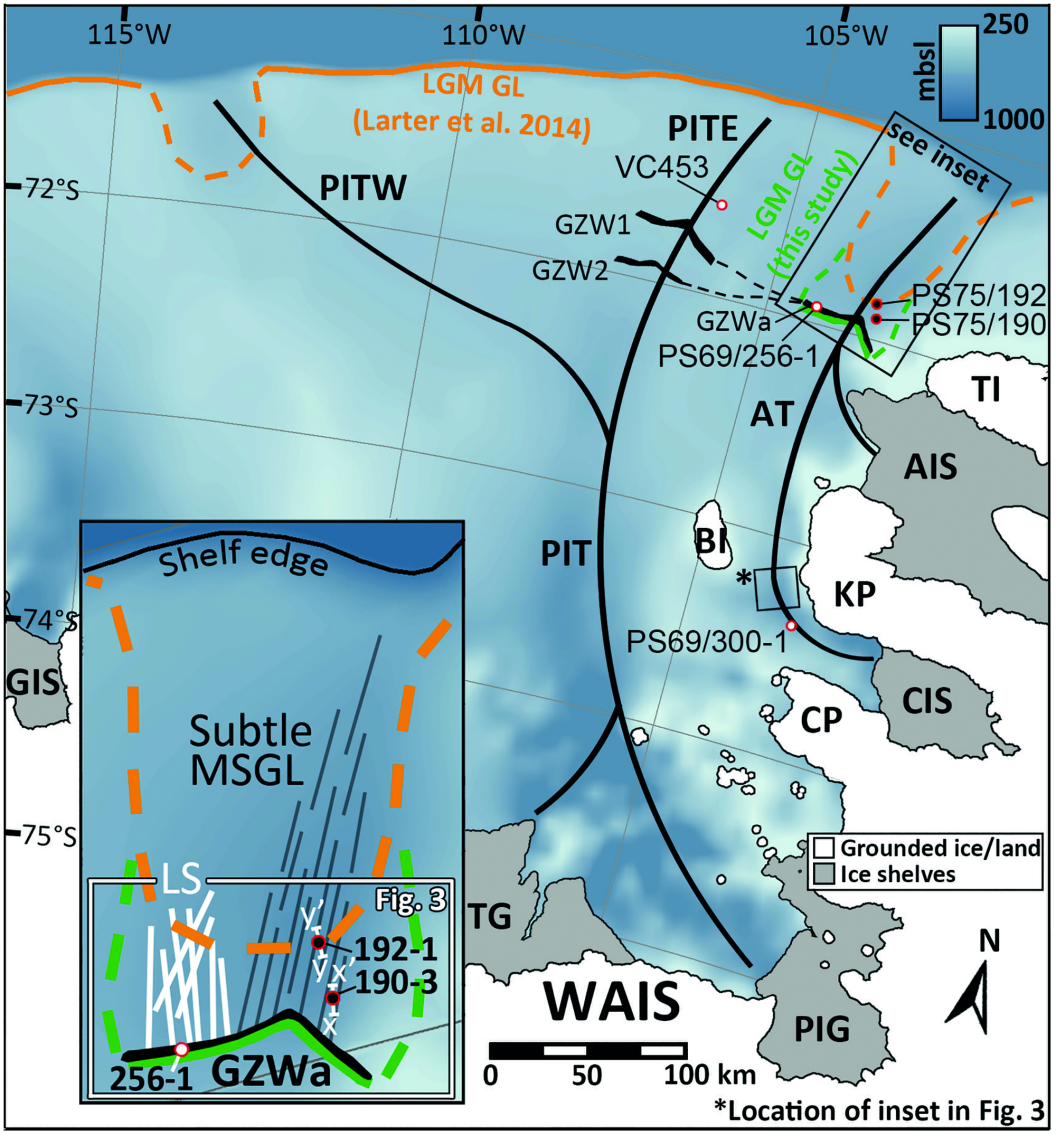
Map of the eastern Amundsen Sea Embayment, West Antarctica. The location of the study area is indicated by large black box in the upper right corner. The general bathymetry is based on IBCSO v. 1 data [27]. Continuous orange line indicates the LGM grounding line position (25 ka BP; [23])–dashed sections mark uncertain positions. Continuous green line indicates the LGM grounding line position based on this study—dashed green line marks yet uncertain sections. Locations of grounding-zone wedges (GZW) derived from Graham et al. [8] (GZWs 1 & 2), and Klages et al. [13] (GZWa). Thick black lines mark the axes of paleo-ice stream troughs. Locations of sediment cores described in detail for this study are indicated by red-circled black dots (cores VC453, PS69/256-1, and PS69/300-1 [11] are indicated by red-circled white dots). Lines x-x’ and y-y’ in inset mark the location of PARASOUND profiles in Fig 4. The simplified display of mega-scale glacial lineations (MSGLs), linear scours (LS), and the grounding-zone wedge ‘a’ (GZWa) in the white box in inset is based on glacial landform information presented in Fig 3, and from recent reconstructions by Klages et al. [13]. Small black box indicates the location of MSGLs shown in the inset in Fig 3. WAIS––West Antarctic Ice Sheet; PIT(W, E)––Pine Island-Thwaites Trough (West, East); AT––Abbot Trough; BI––Burke Island; TI––Thurston Island; AIS––Abbot Ice Shelf; KP––King Peninsula; CIS––Cosgrove Ice Shelf; CP––Canisteo Peninsula; PIG––Pine Island Glacier; TG––Thwaites Glacier; mbsl––meters below sea level.

Detailed bathymetry data by Klages et al. [13] revealed that this trough is an unusually overdeepened basin with a suite of clearly identifiable ice-marginal landforms, unmodified by iceberg scours and, critically, undisturbed sedimentary sequences. However, the bathymetry alone was not sufficient to determine the age of these features and to define if this trough had been covered by grounded ice during the last LGM. Previous work in Pine Island-Thwaites Trough and Abbot Trough inferred the LGM grounding line located somewhere between the continental shelf edge (maximum limit) and the first clearly identified subglacial bedforms just further inland (minimum limit) [8,23]. Grounding-zone wedges (GZWs) in the outermost sections of both Pine-Island Thwaites and Abbot Trough (‘GZW1’ and ‘GZWa’, Fig 2) are interpreted to indicate prolonged halts of the grounding line either during or after the last glacial period [8,13] rather than the terminus of an expanded ice sheet, but only a few attempts have yet been made to date these features [10,13,24]. Further to the west, no large GZWs or moraines have been mapped in the outer Dotson Getz Trough. Larter et al. [25] interpreted this as evidence for the grounding line reaching the shelf edge here during the LGM. However, sedimentological evidence and age constraints are similarly lacking for this part of the embayment.

Here we present the first direct marine geological and geophysical evidence from the Amundsen Sea Embayment showing that the WAIS did not reach the continental shelf edge everywhere in this critical sector during the last glacial period. This finding is significant as it redefines the shelf limit for the LGM-ice sheet in this area, which is important to help defining its last maximum extent and subsequent retreat history, and also questions about the source of postglacial sea-level rise. Further, this grounded-ice free area may have also served as a glacial refuge for Antarctic shelf benthos, where it could survive the harsh conditions during the last glacial period.

## Materials and methods

**Fig 3:**
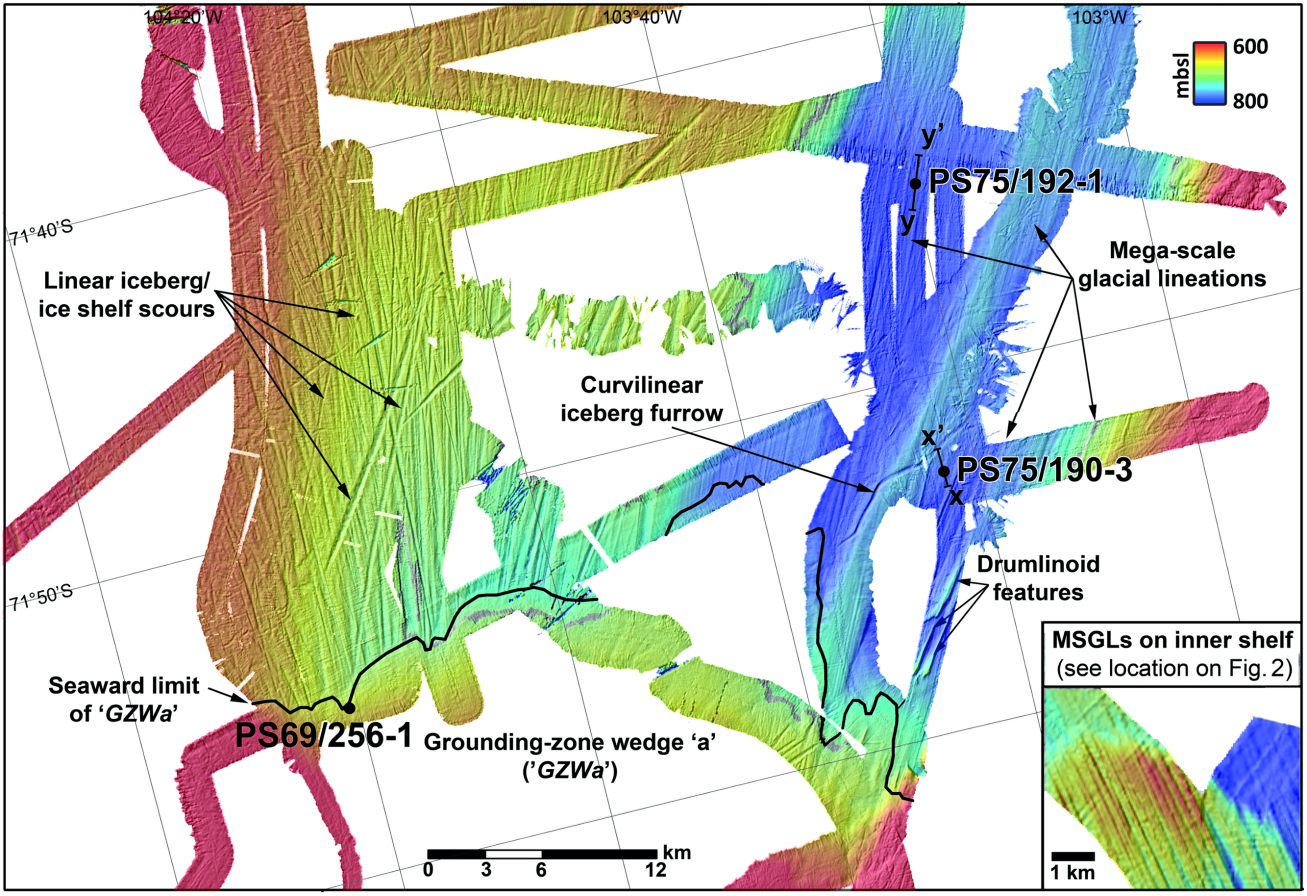
Detailed multibeam swath bathymetry. Data shows the location of grounding-zone wedge ‘a’ (‘GZWa’) and glacial landforms emerging NNE from it within the deep (~800 meters below sea level) outer portion of Abbot Trough. Sediment core locations are indicated by black dots, sediment echography profiles in Fig 4 are marked by lines x-x’ and y-y’. Inset shows MSGLs located in small black box in Fig 2. Grid cell size 30 m, grid illuminated from NW.

The bathymetry presented in detail here (Fig 3) formed part of a wider compilation recently published by Klages et al. [13]. The sediment cores and sediment echography data used in this study were collected on the outer Amundsen Sea Embayment (ASE) shelf during RV Polarstern expeditions ANT-XXIII/4 (PS69) in 2006 and ANT-XXVI/3 (PS75) in 2010 [29,30]. To characterize the sub-seafloor sediments in the deep outer shelf part of Abbot Trough, we profiled the seabed using PARASOUND, a parametric 4 kHz sediment sub-bottom profiler. Two profiles crossing the core locations were analyzed (Figs 2 and 3). During expedition PS75 two gravity cores from PS75 (PS75/190-3 and PS75/192-1) were recovered from an overdeepened (~800 mbsl; meters below sea level) outer shelf section of Abbot Trough (Fig 1) which, with one exception, was unaffected by iceberg scouring (see Fig 3).

The two sediment cores, and core PS69/256-1 which had been previously collected during expedition PS69 from the toe of ‘GZWa’ further south (Figs 2 and 3; [11]), were sampled and analyzed following a standard multi-proxy approach, including the determination of grain sizes, magnetic susceptibility, water contents, shear strengths, P-wave velocity, and organic carbon (C_org_) content (e.g. [12]). Combined with sedimentary structures, lithological composition, and grain-size distribution core facies were characterized. Additionally, palaeomagnetic intensities were measured on core PS75/190-3. All cores contained sufficient amounts of calcareous microfossils for AMS ^14^C dating. These are rare in sediments from the Antarctic continental margin (e.g. [31]) but provide the most reliable ^14^C age control for Antarctic shelf sediments deposited during the last ca. 40 ka. The low amounts of calcareous microfossils were dated in the MICADAS accelerator mass spectrometry radiocarbon dating facility at the Swiss Federal Institute of Technology (ETH) in Zürich, which is especially suitable for dating very small carbonate sample sizes (~80–250 μg CaCO_3_; [32]). We corrected all ^14^C dates by subtracting the marine reservoir effect for Antarctic shelf sediments (1300±100 yrs; [33]), which is in accordance with an uncorrected seafloor surface sediment age of 1320±58 ^14^C yrs from core PS75/192-3 (see Table 1). All ^14^C dates were calibrated with the Calib 7.1 software [34].

**Table 1:** Accelerator mass spectrometry radiocarbon ages. Included information: Core number, publication code, sampling depth, dated material [pF = planktic foraminifera; bF = benthic foraminifera], radiocarbon age [yrs BP], standard deviation [±1σ], marine reservoir effect [MRE], calibrated years [yrs] before the present [BP].

Core	Lab code	Depth (cmbsf)	Material dated	^14^C age (yrs BP)	±1σ	MRE	Calibrated yrs BP (Calib 7.1)	± Range
PS75/190-3	ETH-62113	1	pF, bF	3673	57	1300	2515	187
PS75/190-3	ETH-62112	10	pF, bF	6384	96	1300	5868	256
PS75/190-3	ETH-62111	70	pF, bF	16187	343	1300	18038	796
PS75/190-3	ETH-62110	200	pF, bF	40264	2156	1300	42731	3956
PS75/190-3	ETH-64663	200	pF, bF	31690	1054	1300	34141	2303
PS75/190-3	ETH-55027	287	pF, bF	42819	3076	1300	>45000	/
PS75/192-1	ETH-62117	1	pF, bF	2495	75	1300	1128	179
PS75/192-1	ETH-62115	70	pF, bF	14053	357	1300	15059	1098
PS75/192-1	ETH-64667	160	pF, bF	36514	1509	1300	39261	3006
PS75/192-1	ETH-64665	214	pF, bF	30199	1146	1300	32937	2081
PS75/192-1	ETH-55026	214	pF, bF	36240	1866	1300	38901	3598
PS75/192-3	ETH-61264	0–1	pF	1320	58	1300	0	/
PS75/192-3	ETH-61265	10	pF	6380	55	1300	5841	189
PS69/256-1	ETH-62261	10	pF, bF	7880	95	1300	7469	192
PS69/256-1	ETH-62262	55	pF, bF	13217	142	1300	13778	338

In order to ensure a robust core chronology, additional samples were taken from core PS75/190-3 for palaeomagnetic investigations. The intensity of the Earth's magnetic field undergoes time dependent changes and may be estimated from the natural remanent magnetization (NRM) of marine sediments. Samples for these intensity measurements were taken side by side downcore from core PS75/190-3 with 2.2 cm×2.2 cm×1.8 cm plastic cubes resulting in a spatial resolution of about 2.3 cm. Discrete samples were analysed in the palaeomagnetic laboratory at the Faculty of Geosciences, University of Bremen. Palaeomagnetic directions and intensities of NRM, anhysteretic remanent magnetization (ARM) generated in a peak alternating field of 100 mT and a biasing DC field of 50 μT were measured on a cryogenic magnetometer (2G Enterprises model 755 HR). NRM was measured on each sample before it was subject to a systematic demagnetization treatment involving 15 steps for each sample applying 5 mT increments up to an alternating field of 50 mT and 10 mT increments in alternating fields between 60 and 100 mT. A detailed vector analysis was applied to the results [35] in order to determine the characteristic remanent magnetization (ChRM). The mean maximum angular deviation (MAD) for ChRM is 5.7° ranging from 1.2° to 15.8°, indicating a reasonably well-defined magnetization component [36]. Inclination of the ChRM of core PS75/190-3 oscillates from -87° to +58° with a mean inclination of -64.3° (α_95_ = 8°), matching the present day inclination of -67.15° at the core site according to the International Geomagnetic Reference Field (IGRF-12) [37]. Relative palaeointensity (RPI) estimates were calculated using the so-called ‘slope-method’ or pseudo Thellier method [38,39]. RPI was computed as the slope of the regression line of NRM intensities plotted versus the intensities of ARM for AF demagnetization levels 15 to 40 mT (NRM15-40mT / ARM15-40mT (RPI_ARM(15-40mT)_)). The results were standardized as zero mean with standard deviation 1.

The sediment core data presented here can be obtained from the PANGAEA data repository (https://doi.org/10.1594/PANGAEA.857881).

## Results and interpretations

### Seabed geomorphology and sub-seafloor stratigraphy

Landforms indicative of the maximum extent of grounded ice on the outer shelf were mapped in Abbot Trough with multibeam echosounder data (Fig 3). The most prominent feature is a sediment wedge with a curving, chevron-shaped NE-facing slope. The wedge is interpreted as an ice-stream grounding line deposit, a grounding zone wedge (GZW), and was referred to previously as ‘GZWa’ [13]. The landform indicates a past and prolonged halt of the grounding line at this location. In its western part, ‘GZWa’ clearly overlies NNE-ward trending linear scours. In the eastern part, NE-ward trending parallel mega-scale glacial lineations (MSGLs) emerge from beneath the wedge ([13]; Fig 3). The MSGLs indicate the former grounded signature of an ice stream beyond, thus pre-dating, the wedge formation (e.g. [40]). The morphometry of these MSGLs is notably subtler when compared to similar subglacial bedforms imaged farther inland (see inset in Fig 3). Furthermore, the MSGLs have been unaffected by erosion, likely because they are located within the unusually deep outer portion of Abbot Trough (~800 mbsl) at water depths below maximum iceberg-keel draft, which protected them from scouring by iceberg keels. Whilst the overall appearance of the lineations cannot provide a direct constraint on their age, it nevertheless implies formation under different conditions and possibly during a WAIS advance pre-dating the LGM (cf. [8]).

**Fig 4:**
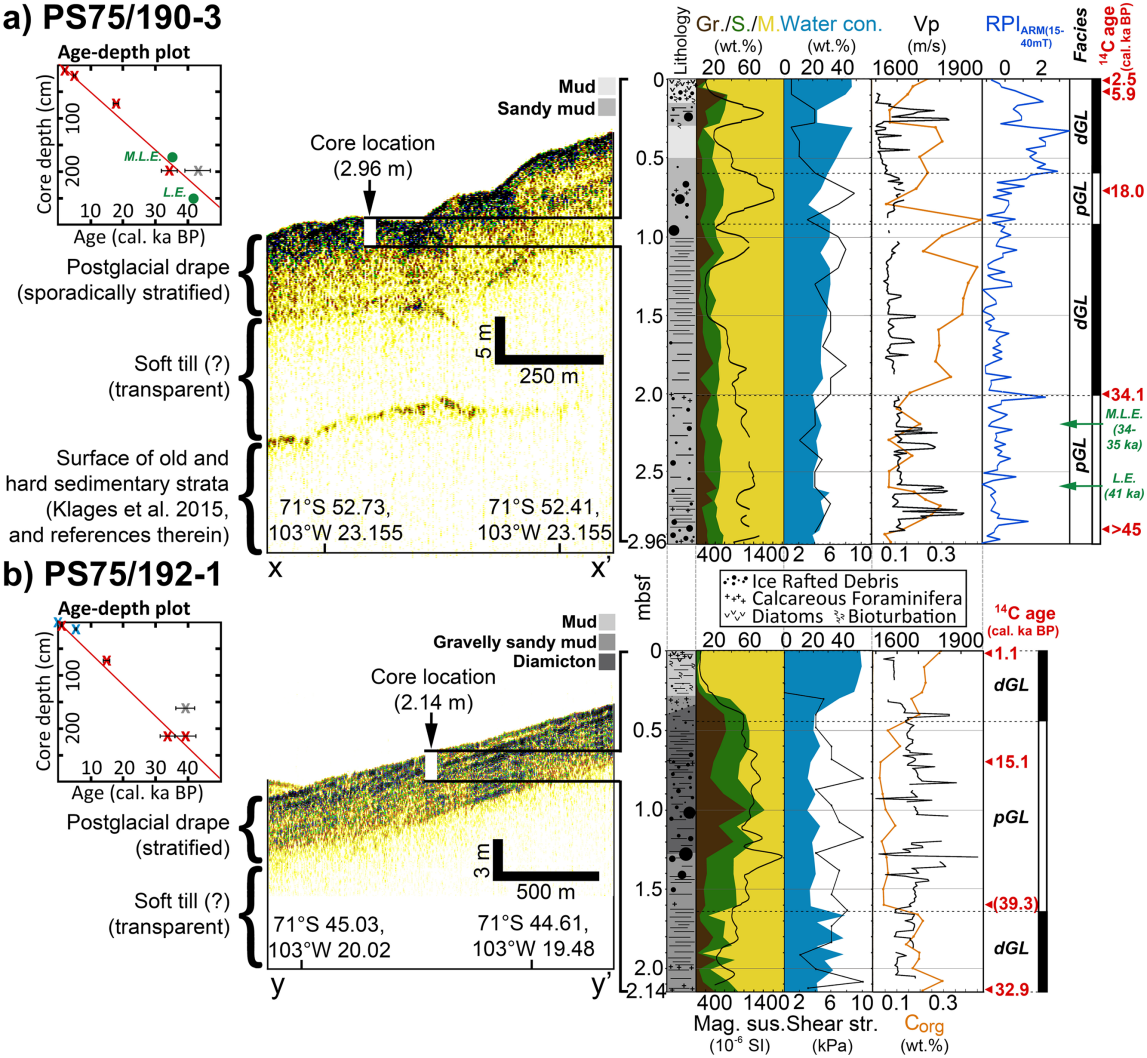
Sediment echography profiles and data logs for sediment cores PS75/190–3 and PS75/192-1. The cores were recovered from the upper parts of an acoustically stratified unit that overlies an acoustically transparent unit. Core data include lithology (lithological key is given above respective subbottom profiles), grain-size distribution (gravel/sand/mud), magnetic susceptibility, water content, shear strength, p-wave velocity (Vp), organic carbon content (C_org_), relative palaeointensity (RPI_ARM(14-50mT)_) in standardized form (only for PS75/190-3 –M.L.E. = ‘Mono Lake Excursion’; L.E. = ‘Laschamp Excursion’), facies (described in text), and calibrated (cal.) accelerator mass spectrometry radiocarbon ages of mixed benthic/planktic calcareous foraminifera in kiloyears before present (ka BP). The age models for the cores are displayed in boxes ‘Age- depth plot’ (red crosses refer to ages obtained from gravity cores PS75/190-3 & PS75/192-1; blue crosses: PS75/192-3; age uncertainties are indicated by black bars; dates with asterisk indicate duplicate dates for samples PS75/190-3 200 centimeter below seafloor (cmbsf) and PS75/192-1 214 cmbsf; stratigraphic locations of M.L.E. and L.E. are indicated by green dots).

In order to provide an explanation for the differences in MSGL appearance, PARASOUND profiles from seaward of ‘GZWa’ were investigated. They reveal a crudely to strongly stratified acoustic unit that conformably blankets an underlying acoustically transparent unit (Fig 4). The latter likely corresponds to a soft till, into which the MSGLs were moulded (S1 Fig), as it has been observed in numerous other palaeo-ice stream troughs on the Antarctic shelf (e.g. [6,8,10]). Neither of these units was detected in reflection seismic profiles acquired from within Abbot Trough (e.g. [41]), but the upper draping unit is unusually thick for this location, distal to the ice sheet. The drape corresponds to at least 9 meters (and potentially up to 12 m) of sediment cover overlying the bedforms (Fig 4; S1 Fig). This contrasts to only ~80 cm of glaciomarine cover above a subglacial till in core PS69/300-1 (see location in Fig 2) that corresponds to the surface of inner shelf MSGLs (see Fig S1h in Smith et al. [11]). The weaker reflectivity in the upper profile across site PS75/190-3 (Fig 4a) is likely due to stronger scattering of energy caused by a different survey orientation over a more rugged topography, when compared to site PS75/192-1 (Fig 4b).

### Sedimentary facies

Two cores (PS75/190-3 and PS75/192-1) were recovered from the upper part (Figs 2–4) of the thick and stratified acoustic unit in order to identify its composition and constrain its age. Both cores contain unconsolidated, slightly to strongly laminated/stratified, gravel-bearing hemipelagic muds with rare but intact shells of both planktic and benthic foraminifera, medium to high water contents between 20–50 wt.%, and generally low shear strength values of <10 kPa (Fig 4; S2 Fig). These sediment properties suggest deposition in an ice-proximal to ice-distal glaciomarine environment without indication for sediment compaction or reworking by grounded ice or iceberg keels (e.g. [10,11,14,42]). Predominantly fine-grained, homogenous to slightly stratified hemipelagic sediments with scattered larger pebbles and traces of well-preserved planktic and benthic foraminifera shells, variable magnetic susceptibility (MS) (0–1000 10^−6^ SI), low shear strengths (on average 5 kPa) and P-wave velocities (Vp) (~1500–1600 ms^-1^), but high water and C_org_ contents (0.2–0.4 wt.%) suggest a low-energetic seasonal-open marine environment distal to the grounding line (Facies dGL), characterized by the alternating settling of hemipelagic and meltwater plume material (e.g. [42]), and the occasional release of ice-rafted debris (IRD) from the base of drifting icebergs. Coarser sediments characterized by slight to strong stratification and higher pebble contents, higher MS (>1000 10^−6^ SI), medium shear strength (on average 7 kPa), lower water (15–20 wt.%) and C_org_ contents (<0.1–0.2 wt.%), and a generally high Vp (1600–2000 ms^-1^) likely record meltwater flows, current winnowing of sediments, or sub-ice shelf rain-out, and thus are attributed to a higher-energetic environment more proximal to the grounding line (Facies pGL) (Fig 4) (e.g. [42]).

The pGL-facies in core PS75/192-1 is characterized by a prominent interbedded stratified diamicton, which contains traces of intact mixed planktic and benthic foraminifera shells, and likely corresponds to the distinct uppermost subbottom reflector in the PARASOUND profile (Fig 4b), thus suggesting that the weaker reflections underneath indicate previous grounding line advances proximal to the core location. The reflections throughout the stratified unit (Fig 4b) exclude the presence of a thick transparent layer near the seafloor, usually corresponding to a massive, terrigenous subglacial till (e.g. [43]). Therefore, we are confident that the stratified acoustic unit consists entirely of glaciomarine sediments as recovered in the cores. These sediments therefore have likely been deposited after the last coverage with grounded ice, which in turn seems to be recorded by the MSGLs over which these deposits now lie (Fig 4; S1 Fig).

### Radiocarbon chronology and palaeomagnetic age constraints

#### Radiocarbon chronology

In contrast to the majority of sediment cores from the outer ASE continental shelf [10,14], sedimentary structures in both cores—entirely comprising glaciomarine sediments—do not show any indication for reworking or turbation associated with seafloor-scouring by iceberg keels (“Lithologies” in Fig 4; S2 Fig). Instead and in contrast to the sediment characteristics Smith et al. [10] presented for the identification of iceberg turbates, the sediments investigated here are slightly to strongly stratified, and have—in comparison—low magnetic susceptibility and shear strength values, without showing any significant variations. This is in accordance with our bathymetry data (Fig 3) not revealing any scour marks on the seafloor in the deep outer Abbot Trough. Hence, they record primary deposition and therefore serve as reliable recorders of past regional WAIS change. Well-preserved calcareous foraminifera were extracted from each facies for radiocarbon dating (Fig 4; Table 1). With the exception of two pairs of replicate ages that returned different ^14^C dates at 200 centimeter below seafloor (cmbsf) in core PS75/190-3 and 214 cmbsf in core PS75/192-1, and one significantly older age at 160 cmbsf in core PS75/192-1, all radiocarbon ages in both cores occur in stratigraphic order (Fig 4; Table 1), or overlap within the analytical error (PS75/192-1; 214 cmbsf). We dismiss the nearly radiocarbon-dead age at 200 cmbsf in core PS75/190-3 (42.7 cal. ka BP) on the basis of the palaeomagnetic stratigraphy (Fig 4a; see below) and attribute the discrepancy between the two replicate dates and the old age at 160 cmbsf in core PS75/192-1 to larger analytical uncertainty for samples older than 30 ka, as well as larger uncertainties associated with small carbonate sample sizes, particularly in case of the old age at 160 cmbsf in core PS75/192-1, which only provided 14 μg of carbon to be measured. We rather exclude the possibility of recycling of ‘older’ foraminifera from deeper horizons, since they mostly occur in moderately to strongly stratified unconsolidated sediments without noticeable bioturbation marks below 30–40 cmbsf (Fig 4; S2 Fig), thus clearly indicating hemipelagic deposition in a glaciomarine environment. Moreover, the relatively constant age-depth relationships in both cores (inset ‘Age-depth plots’ in Fig 4) provide no evidence for a significant hiatus in sedimentation at these sites over the past ~40.000 years.

In addition, we dated calcareous microfossils in sediments from core PS69/256-1 from the toe of ‘GZWa’. Recently, Smith et al. [11] reported that core PS69/256-1 records the succession from a grounding line-proximal glaciomarine diamicton (Facies 1b) via a more grounding line-distal transitional sandy-gravelly mud unit (Facies 2) to grounding line-distal hemipelagic glaciomarine muds (Facies 3) (see Fig 2 in Smith et al. [11]). They reported a deglacial age (dating the acid insoluble organic (AIO) fraction) of ~17 cal. ka BP [11]. We re-sampled the core and were able to date calcareous microfossils from both their Facies 3 (10 cmbsf: 7.5 cal. ka BP) and from the base of their Facies 2 (55 cmbsf: 13.8 cal. ka BP) (Table 1). Our new age, which is about 3.000 years younger than the previously reported AIO date, could imply that grounded ice retreated later from GZWa than previously thought, although additional age-data are required in order to confirm this.

#### Palaeomagnetic age constraints

**Fig 5:**
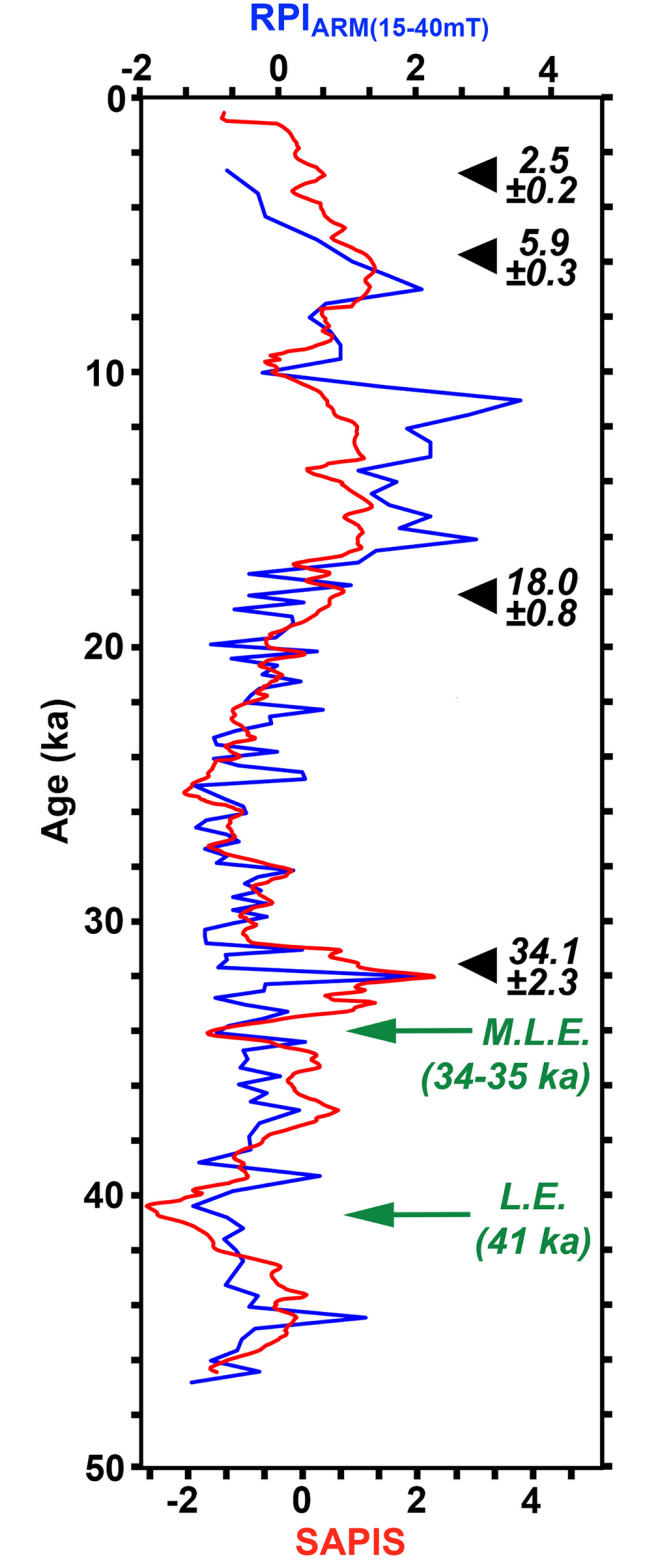
Relative palaeointensity. Relative palaeointensity record (NRM(demag15-40mT)/ARM(demag15-40mT)) for core PS75/190-3 (blue) plotted besides SAPIS palaeointensity stack (red) [45], including the radiocarbon ages (black arrows), and stratigraphic locations of the Mono Lake Excursion (M.L.E.) and Laschamp Excursion (L.E.) (green arrows). Both data sets are shown in standardized form.

Because of increasing radiocarbon dating uncertainties towards the bottom of both cores, we applied palaeomagnetic measurements in order to test the ^14^C chronology. However, we were only able to measure palaeomagnetic directions and intensities through core PS75/190-3 (Fig 4a) and had to disregard core PS75/192-1 for these measurements because of its heterogeneous lithological composition, expressed by large variations in grain-size (Fig 4b), which would have likely affected and altered the palaeomagnetic signal. The correlation of the relative palaeointensity (RPI) estimate of core PS75/190-3 (Fig 5) to the South Atlantic geomagnetic palaeointensity stack (SAPIS) [44] resulted in a correlation coefficient (Pearson’s ‘r’) of r = 0.68, thus resolves the uncertainty of radiocarbon age constraints (30–40 cal. ka BP) from the lower 1.5 m in core PS75/190-3, and extends the chronological control for this core. Considering the radiocarbon chronology, and correlation to the SAPIS stack, the RPI lows at 220.4 cmbsf and 257.4 cmbsf in core PS75/190-3 may be attributed to the ‘Mono Lake Excursion (MLE)’ (~34–35 ka BP [45]) and the ‘Laschamp Excursion (LE)’ (~41 ka BP [46]), respectively (Fig 5). Although records of Late Quaternary geomagnetic excursions from the Southern Hemisphere and thus from the Southern Ocean are still sparse, Lund et al. [45] and Cassidy [47] reported evidence for the MLE, while Collins et al. [48] identified the LE in Antarctic marine sediments. Recently, Xiao et al. [49] found both MLE and LE recorded in marine sediments from the Scotia Sea.

## Maximum extent of grounded ice and implications

Our results demonstrate that glaciomarine sedimentation must have prevailed in outer Abbot Trough before the LGM, since sediments from the up to 12 m-thick glaciomarine drape are already more than 40 cal. ka BP old in the upper 2–3 meters (Fig 4). This conclusion is supported by both radiocarbon dating and palaeomagnetic data. Consequently, the grounding line must have been situated south of the locations of both PS75/190 and PS75/192 over this time period. The simplest interpretation of the prominent ‘GZWa’ south of the core sites (Figs 2 and 3) is that it records the LGM position of the grounding line ~100 km landward of the shelf edge. The implication of this finding is that a significant area of outer shelf seafloor, at least 6000 km^2^ in size, was free of grounded ice for at least the last 41,000 years (Fig 2). The unusual depth of outer Abbot Trough—when compared to other outer ASE troughs (e.g. PIT [8] or DGT [6])–seemed to have played a major role in preventing local ice grounding here. While ice may have been just thick enough to ground in outer PIT and DGT and in the shallower vicinity of the outer Abbot Trough—which remains to be tested—it was probably too thin to ground on the seafloor in outer Abbot Trough. The unusual depth here has likely been the main reason for leaving bedforms and sediments from previous glacial maxima largely undisturbed here. The date of 13.8 cal. ka BP from the transitional sediments in core PS69/256-1 remains subject to some uncertainty, but our new age, together with previously published data [11] suggests that the grounding line retreated from ‘GZWa’ sometime between 17.0 and 13.8 cal. ka BP. Additionally, the two cores from outer Abbot Trough contain sediments with a pGL-facies at similar sub-bottom depths whose upper parts yielded ages of 15.1 and 18.0 cal. ka BP, respectively (Fig 4). These ages are consistent with an LGM termination at ‘GZWa’. When the grounding line advanced towards the location of ‘GZWa’ at some point before 18 cal. ka BP, it must have paused and accumulated subglacial till to build up the wedge. At the same time meltwater and debris flow deposits, and material from sub-ice shelf rain-out were deposited just seaward of the wedge.

Our calculated ~6000 km^2^ grounded-ice free shelf area is a conservative estimate. ‘GZW1’ in neighboring PIT may be a correlative of ‘GZWa’ based on corresponding geographic locations and similar sizes (Fig 2) (cf. [13]). If ice did not advance beyond ‘GZW1’ at the LGM, then a much larger proportion of submarine shelf area must have remained grounded-ice free during the last glacial period. In PIT, Smith et al. [11] suggested that grounding line retreat from the outer shelf must have commenced around 20.6 cal. ka BP (core VC453; Fig 2). However, the corresponding AIO age was obtained from the top of a diamicton, which could not be attributed confidently to subglacial deposition as the surrounding area had been intensely scoured by iceberg keels, thus the diamicton may be an iceberg turbate rather than a subglacial till [11]. Indeed, iceberg turbation on shallow outer shelf portions remains one of the biggest problems for retrieving undisturbed sediments. In order to establish reliable chronologies, it is important that future work helps identify and then sample areas unaffected by such processes.

Our data serve as the first direct empirical evidence that the grounding line in the eastern ASE did not advance to the shelf edge during the LGM. Therefore we provide direct constraints on extents that have only previously been inferred and hypothesized (e.g. [8,23]). Hence, our results corroborate and add to previous results from the western Ross Sea [17,26], offshore George V Land [19], and Prydz Bay [18,50], where limited LGM grounded ice extents have also been shown (Fig 1).

A further significant implication of our data is that our cores penetrated and recovered only the upper one third of a more expanded, thick glaciomarine sequence. We suggest on the basis of our combined radiocarbon-palaeomagnetic chronology from the uppermost part of the postglacial drape that the entire thickness of the drape (Fig 3) documents the prevalence of grounded-ice free conditions for much longer than this. Indeed, based on sedimentation rates for our cores, it is possible that the shelf has remained free of grounded ice since glacial Marine Isotope Stage 6 (MIS6) (191–130 ka). This would, in turn: (1) exclude the possibility of an early pre-LGM retreat from a shelf edge position; (2) suggest that some sea-floor bedforms (the subtle MSGLs underlying the glaciomarine drape) formed during a preceding glacial maximum (Fig 3), and (3) importantly, suggest the potential for the existence and recovery of last interglacial marine sediments on the West Antarctic shelf. Considering the lack of foraminifera in the cores during the LGM-period (34.1–18.0 cal. ka BP in core PS75/190-3 and 32.9–15.1 cal. ka BP in core PS75/192-1), as well as the absence of bioturbation below 30–40 cmbsf in both cores, the presence of an ice shelf seawards of ‘GZWa’ during this time may be plausible. In support, Dowdeswell and Fugelli [51] stated that the presence of GZWs is normally associated with an ice shelf extending seawards. Although our cores do not provide direct evidence that the outer shelf part of Abbot Trough acted as a refuge for Antarctic shelf benthos during the last glacial period, we point out that the presence of an ice shelf does not exclude this scenario. Benthic life could have survived either seaward of the ice-shelf edge or even elsewhere under the ice shelf. Studies from the modern Amery Ice Shelf in East Antarctica have demonstrated that phytoplankton particles produced in seasonal open water offshore from the ice-shelf front can be advected by currents up to 100 km underneath the ice shelf and sustain a rich benthic community [52–54]. Thereby, the distribution and abundance of the benthic organisms strongly depends on the inflow pathway of the currents [53,54]. Although it is assumed that in the Amundsen Sea the waters north of the shelf edge were permanently sea-ice covered at the LGM [55], polynyas may have opened up at least episodically offshore from the ice shelf covering outer Abbot Trough, and enabled phytoplankton production (cf. [56]). Indeed, geological evidence for the existence of glacial-time polynyas over the Amundsen Sea continental slope has been presented previously [56,57].

Ultimately, there is ongoing debate surrounding whether parts of the Antarctic shelf acted as refugia for benthic organisms during the LGM (e.g. [16]), or whether habitats shifted to the Antarctic continental slope or sub-Antarctic islands to escape the harsh glacial conditions (e.g. [15,58]). Our results now provide robust geological evidence for a grounded-ice free outer Abbot trough, by which testing the shelf refugia hypothesis may be possible in a much more focused way for the Amundsen Sea region.

In conclusion, our data provide new constraints on the LGM ice-sheet position in the Amundsen Sea, and serve as the first geological evidence of a limited GL advance onto the Amundsen Sea shelf. This information will improve regional palaeo-ice sheet reconstructions, and may also provide another piece of evidence for calibrating and evaluating palaeo-ice sheet models, which are used to simulate past and future WAIS configurations. It further adds to the list of potential shelf refugia for Antarctic benthos during the last glacial period.

## Supporting information

S1 Fig Sediment echography profiles. These profiles are perpendicular to profiles x-x’ and y-y’ in Figs 2 and 3 showing postglacial sediments draping mega-scale glacial lineations (MSGLs). Insets indicate the leveling of the initial MSGL relief by glaciomarine sediments. The weak reflectivity in comparison to Fig 4b is likely due to stronger scattering of energy caused by a different survey orientation over a more rugged topography.
(TIF)

S2 Fig Linescan photographs and corresponding X-radiographs of cores PS75/190-3 and PS75/192-1. Zoomed-in examples for degree of lamination/stratification are included.
(TIF)
