# Supplementary figures and images for: Limited grounding-line advance onto the West Antarctic continental shelf in the easternmost Amundsen Sea Embayment during the last glacial period
Source: PLoS One. 2017 Jul 25;12(7):e0181593. doi: 10.1371/journal.pone.0181593 (PMC5526568; doi:10.1371/journal.pone.0181593)

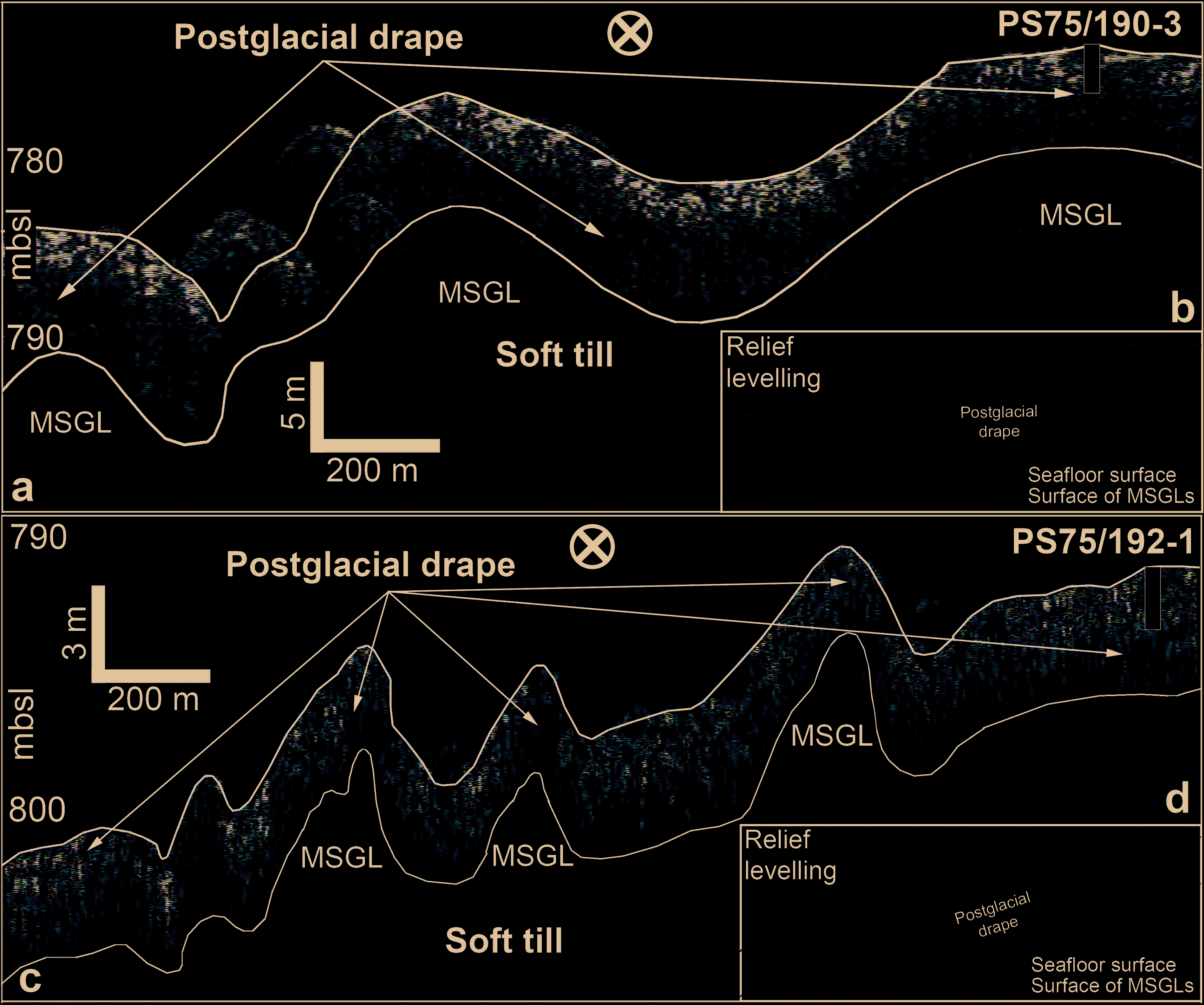

Supplement: S1 Fig — These profiles are perpendicular to profiles x-x’ and y-y’ in Figs 2 and 3 showing postglacial sediments draping mega-scale glacial lineations (MSGLs). Insets indicate the leveling of the initial MSGL relief by glaciomarine sediments. The weak reflectivity in comparison to Fig 4b is likely due to stronger scattering of energy caused by a different survey orientation over a more rugged topography. (TIF) [file pone.0181593.s001.tif]

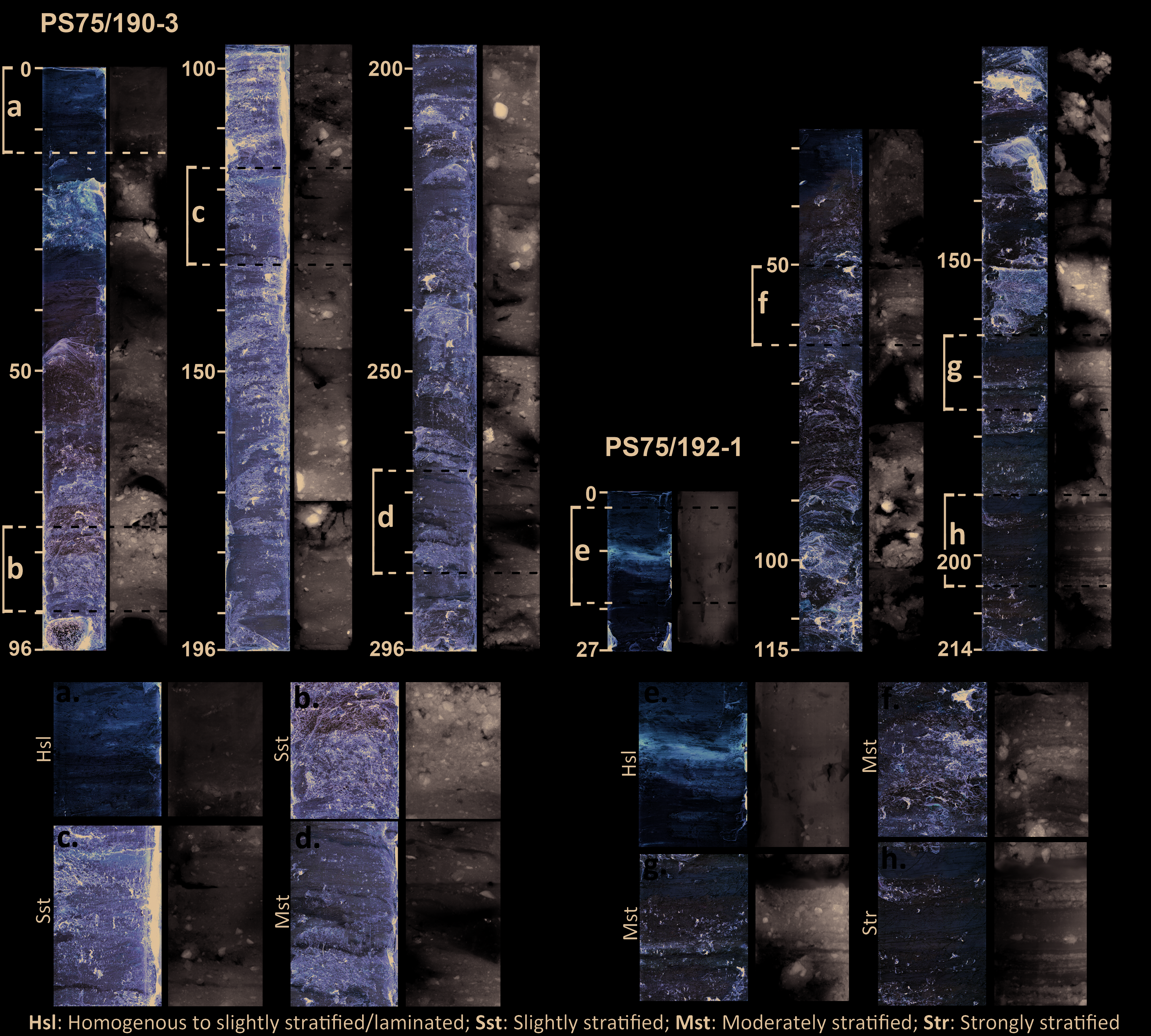

Supplement: S2 Fig — Zoomed-in examples for degree of lamination/stratification are included. (TIF) [file pone.0181593.s002.tif]
